# A High Fuel Consumption Efficiency Management Scheme for PHEVs Using an Adaptive Genetic Algorithm

**DOI:** 10.3390/s150101245

**Published:** 2015-01-12

**Authors:** Wah Ching Lee, Kim Fung Tsang, Hao Ran Chi, Faan Hei Hung, Chung Kit Wu, Kwok Tai Chui, Wing Hong Lau, Yat Wah Leung

**Affiliations:** 1 Department of Electronic and Information Engineering, Hong Kong Polytechnic University, Hung Hom, Kowloon, Hong Kong, China; E-Mail: enwclee@polyu.edu.hk; 2 Department of Electronic Engineering, City University of Hong Kong, Kowloon Tong, Kowloon, Hong Kong, China; E-Mails: haoranchi2-c@cityu.edu.hk (H.R.C.); fhhung4@cityu.edu.hk (F.H.H.); chungkwu4@gapps.cityu.edu.hk (C.K.W.); ktchui3-c@my.cityu.edu.hk (K.T.C.); itwhlau@cityu.edu.hk (W.H.L.); 3 Department of Systems Engineering and Engineering Management, Hong Kong Polytechnic University, Hung Hom, Kowloon, Hong Kong, China; E-Mail: 50997159@student.cityu.edu.hk

**Keywords:** PHEV, adaptive genetic algorithm, fuel efficiency management

## Abstract

A high fuel efficiency management scheme for plug-in hybrid electric vehicles (PHEVs) has been developed. In order to achieve fuel consumption reduction, an adaptive genetic algorithm scheme has been designed to adaptively manage the energy resource usage. The objective function of the genetic algorithm is implemented by designing a fuzzy logic controller which closely monitors and resembles the driving conditions and environment of PHEVs, thus trading off between petrol *versus* electricity for optimal driving efficiency. Comparison between calculated results and publicized data shows that the achieved efficiency of the fuzzified genetic algorithm is better by 10% than existing schemes. The developed scheme, if fully adopted, would help reduce over 600 tons of CO_2_ emissions worldwide every day.

## 1. Introduction

Global warming has gradually become a big issue and it is aggravating because of increasing CO_2_ emissions [1]. In order to minimize CO_2_ and potential pollutants generated by burning fuels, much cleaner alternative energy sources for the next generation of vehicles are desperately sought for [2,3]. Hybrid Electric Vehicles (HEVs) are new cars manufactured for the purpose of generating little or minimum pollutants. A higher HEV fuel efficiency will help to alleviate the carbon release issue. In HEVs, fuel and electric power are available energy sources and may provide energy at the same time [4]. The fuel efficiency of HEV, η_H_, is defined as the percentage of energy consumption due to fuel combustion among the total energy consumed. In particular, in plug-in Hybrid Electric Vehicles (PHEVs), the internal combustion engine is connected directly to the wheels in parallel to the electric motor. Thus, the fuel efficiency of PHEV, η_P_, in general surpasses the respective η_H_. Higher efficiency (η) implies less contamination, rendering a greener environment. A careful review of the existing fuel efficiency schemes reveals that there is still room for improvement of η_P_. Intuitively, if the driving control mechanism conforms to the road conditions, a higher η_P_ will be achieved. In existing PHEV energy consumption schemes, it is observed that under the circumstance of fast speed and high acceleration, more pollutants will be produced than during uniform driving because more fuel is consumed. Thus a more efficient scheme to trade-off fuel-versus-electricity (FVE) is needed. In this communication, an efficient optimization algorithm for PHEV that pertains to road and driving conditions has been developed to achieve the FVE target.

Genetic algorithm (GA) is a good candidate for global optimization [5,6] and former optimizations of η_P_ were attempted [7–9]. It is noted that in former works, vital parameters of PHEVs such as the battery State of Charge (SOC) and the fuel capacity (C), were not considered as key factors in the optimization of η_P_. In this new scheme, the choice of resource consumption between fuel and electricity must take into account the road (e.g., uphill, downhill *etc.*) effect and car condition (e.g., high battery level and/or low fuel level *etc.*). The status of SOC and C thus are indicative of the road and car conditions. In reality, clearcut boundaries must be devised to help decision making for choice of resources. The existing scheme incorporates a fuzzy logic controller (FLC) which fuzzifies the inputs, namely SOC and C. The fuzzification will moderate the objective function of GA. This process is referred as Fuzzified GA (FGA) which is the key contribution in this investigation. The output from the FGA will then drive the PHEV. Such a process will ensure the best η_P_ that pertains to the ever changing road conditions. The fuzzified GA scheme (FGAS) will then trade off FVE. By virtue of the characteristics of FGAS, a plurality of stable objective functions is achieved, rendering a high η_P_. It will be shown from simulated results that FGAS is more efficient than the traditional GA scheme (TGAS).

## 2. Design of TGAS for PHEV

Basic vital parameters that may affect significantly the efficiency of a traditional GAS are listed in Table 1. Settings of the population size and maximum generations conform to the Allele Coverage [10,11]. Such a setting is representative in the design of TGAS.

It was explained that both C and SOC are key parameters of PHEVs. In Table 1, in the ten bits of individuals, the first five binary bits represent the current SOC and the last five bits indicate the current percentage of C. The “SOC” and “C” status divide the whole range [0%, 100%] into thirty-two stages, with intervals of less than 4%, which is sufficient to represent the state of SOC and C accurately.

Uniform crossover with a rate of 0.6 and mutation with a rate of 0.01 are adopted as the main genetic operations based on natural experience [12–14]. Objective functions are vital elements of the TGAS. The objective function, *F_x_*, is shown in Equation (1).

(1) $$\text{MAX}:\;F_{x}=\sum_{i=1}^{10}b_{i}f(i)$$

where *F_x_* is the fitness value of the objective function for the individual *x*, *b_i_* is the binary bit *i* of the individual, *f*(*i*) is the value of *b_i_* and *f*(*i*) is designed as:

(2) $$f(i)=\{\begin{array}{c} 7-i,i\in [1,5]\\ i-11,i\in [6,10] \end{array}$$

The output *F_x_* of TGAS represents η_P_, which is calculated as the ratio of consumed electric power to the total energy consumption.

## 3. Design of FGAS for PHEV

**Figure 1.**
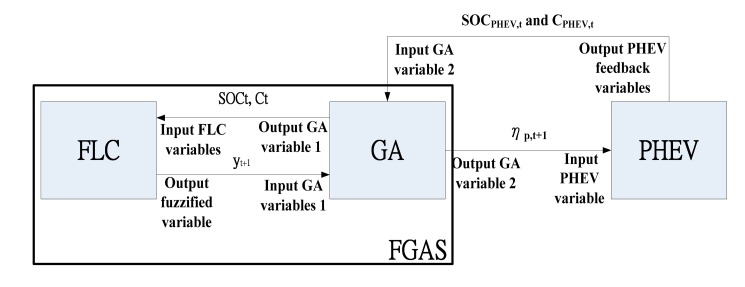
Fuzzified GA scheme (FGAS).

The working principle of FGAS is shown in Figure 1. The initialization values of GA and FGA are the same and are shown in Table 1. It was explained in earlier context that SOC and C from PHEV are representative of road conditions. At time *t*, the PHEV provides output PHEV feedback variables, SOC_PHEV,*t*_ and C_PHEV,*t*_, to the GA. The output GA variable 1, SOC*_t_* and C*_t_*, at the output of the GA are then fuzzified by the FLC. The output fuzzified variable, *y_t_*, moderates the GA variables, SOC*_t_* and C*_t_*, to yield an adaptive η_p,_*_t_*_+1_. The η_p,_*_t_*_+1_ will trade off fuel *versus* electricity for PHEV steering and generate SOC_PHEV,_*_t_*_+1_ and C_PHEV,_*_t_*_+1_ which in turn generate SOC_*t*+1_ and C_*t*+1_. The feedback control process reiterates until the optimal η_P_ is achieved.

## 4. Design of FLC

The fuzzy input variables of FLC, SOC and C, are shown in Table 2. The fuzzification design is categorized into fuzzy sets ranging from “Very low” to “very high” respectively in five levels (SOC_1_-SOC_5_, C_1_– C_5_). The design of fuzzy sets of input FLC variables is shown in Table 2.

The design in Table 2 renders the SOC always has a larger value than C. In the design, the range of SOC*_x_*(*x* = 1,2, …5) is slightly smaller than C*_x_* (*x* = 1,2, …5). For instance, when 80% SOC is classified as “very high” (SOC_5_), the fuel capacity (C_4_) will likely be just “high”. Thus it is seen that with identical proportion of SOC and C, SOC is a stronger indicator than C.

As shown in Table 2, there are twenty five fuzzy rules designed. The format is designed as IF THEN rules, which is shown as “IF SOC is SOC*_x_* and C is C*_x_*, THEN the output FLC variable is Y*_x_*”, where Y*_x_* are the fuzzy sets at the output. Finally, the deffuzified output at the moment *t*, *y_t_*, is calculated by using the centre of area method [9] and given by Equation (3):

(3) $$y_{t}=\frac{\sum_{n}^{x=1}\alpha_{x}(t){\text{Y}}_{x}}{\sum_{n}^{x=1}\alpha_{x}(t)}$$

where *α_x_*(*t*) is strength of the output belonging to the fuzzy sets Y*_x_* at time t (*x* = 1,2,…,5).

The fuzzified output FLC variable, *y_t_*, will then become the input GA variable 1. Attention is drawn to the point that the GA functions of FGAS and GAS are different. In FGAS, the GA function is given by Equation (4):

(4) $$f(i)=\{\begin{array}{c} y_{t}(7-i),i\in [1,5]\\ y_{t}(i-11),i\in [6,10] \end{array}$$

## 5. Simulated Results and Discussion

The design is mainly based on the practical facts of private vehicles in Hong Kong. Under the uniform driving condition, various initial values of SOC and C are used for simulation. The objective is to investigate if FGAS is more adaptive and efficient than TGA in improving η of PHEVs. TOYOTA PRIUS, a popular PHEV in the market, is chosen as a benchmark. Detailed design of the scenarios is described in Table 3.

**Figure 2.**
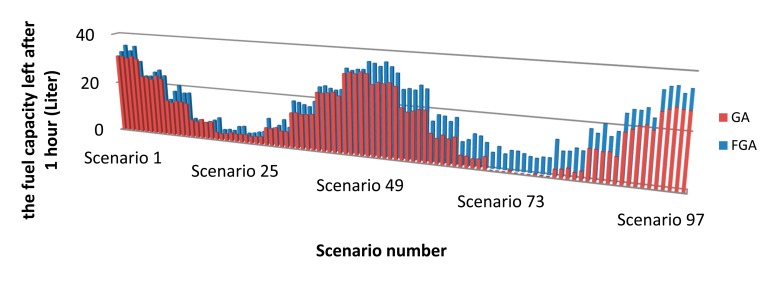
Fuel capacity after one hour (100 scenarios in total).

In the design, 10 states of both SOC and C are used. There will be in total 100 scenarios, which are sufficient to elaborate the practical states of PHEVs. During a 50 km drive in a fluctuating environment, initial values of both SOC and C deviate from the initial state of PHEVs by varying the charge of the battery and fuel level in the fuel tank. It is expected that with an adaptive scheme, a higher accurate and more efficient performance can be achieved. The simulated results of all the 100 scenarios are shown in Figure 2. The average improvement of GA is 8.7%. However, FGA can achieve an improvement of about 18.9%. With unchanged objective functions of TGA, the performance is similar with FGA in those scenarios with high SOC and C. However, in most other scenarios, the premature convergence of TGA occurs frequently. Alternatively, FGA can adjust the objective functions by the fuzzy control action pertaining to the environment to provide a better performance. Therefore, higher fuel efficiency can be achieved by FGA.

As an illustration, the cumulative sales volume of PHEV is more than 7 million units, among which there are more than 4.2 million current users [15]. Based on these figures and from TOYOTA PRIUS-C [16], the average distance that one PHEV travels per day is 28 km which is equivalent to 1 L fuel consumption. It is evidenced that 1 L fuel produces 2.2 kg CO_2_ [17] and that the improvement of FGAS is 10%. As a result, more than 186,880 L of fuel can be saved. This represents a reduction of emissions of CO_2_ by 613 tons every day.

## 6. Conclusions

A fuzzified genetic algorithm (FGA) has been developed to deal with the fuel efficiency management of PHEVs. In FGA, a fuzzy logic controller has been designed to provide control input to the objective functions of genetic algorithm (GA) scheme to ensure FGA be adaptive to the ever changing environment. As a result, the FGA is more efficient than traditional GA (TGA) schemes under unchanged objective functions. Simulated results indicate that FGA has an average improvement of 18.9% while TGA achieves an improvement of 8.7%. Therefore the efficiency of the FGA is 10% better than TGA. With such an improvement, FGA can reduce the CO_2_ emissions by over 600 tons worldwide every day if all PHEVs were to adopt FGA, hence FGAS is a more efficient scheme for the fuel efficiency management of PHEVs.

## Figures and Tables

**Table 1.** GA (FGA) parameters of population and selection.

**GA (FGA) Parameters**	**Designed Data of GA (FGA) Parameters**
Population size	100
Number of maximum generations	100
Number of bits of individuals	10
Selection method	Roulette Wheel Selection (RWS)

**Table 2.** Design of fuzzy sets of inputs of FLC.

**Inputs**	**Function Types**	**Designs (L&U: Lower and Upper Bounds, C: Centre, W: Width of the Top Side of a Trapezoidal Function)**
SOC	Trapezoidal: SOC_1_, SOC_5_	(L,U,C,W) = (−0.2,0.35,0.15,0.15), (0.75,1.2,0.95,0.05)
	Triangular: SOC_2_, SOC_3_, SOC_4_	(L,C,U) = (0.15,0.35,0.55), (0.35,0.55,0.75), (0.55,0.75,0.95)
C	Trapezoidal: C_1_, C_5_	(L,U,C,W) = (−0.1,0.45,0.25,0.25), (0.85,1.3,1.05,0.15)
	Triangular: C_2_, C_3_, C_4_	(L,C,U) = (0.25,0.45,0.65), (0.45,0.65,0.85), (0.65, 0.85,1.05)

**Table 3.** Scenario settings for the comparison of FGAS *versus* TGAS.

**Parameters**	**Designed Data**
Driving condition	Uniform driving
Average speed	50 km/h
SOC (%)	100–10 k (k = 0,1,2, …, 9)
C (%)	100–10 m (m = 0,1,2, …, 9)
Max. capacity of fuel tank	36 Litre
Max. electric power	45 kW
